# Using Corpus Analyses to Help Address the DIF Interpretation: Gender Differences in Standardized Writing Assessment

**DOI:** 10.3389/fpsyg.2020.01088

**Published:** 2020-06-03

**Authors:** Zhi Li, Michelle Y. Chen, Jayanti Banerjee

**Affiliations:** ^1^Department of Linguistics, College of Arts and Science, University of Saskatchewan, Saskatoon, SK, Canada; ^2^Paragon Testing Enterprises, Vancouver, BC, Canada

**Keywords:** writing assessment, gender differences, corpus analysis, linguistic features, differential item functioning, DIF, validation

## Abstract

Addressing differential item functioning (DIF) provides validity evidence to support the interpretation of test scores across groups. Conventional DIF methods flag DIF items statistically, but often fail to consolidate a substantive interpretation. The lack of interpretability of DIF results is particularly pronounced in writing assessment where the matching of test takers’ proficiency levels often relies on external variables and the reported DIF effect is frequently small in magnitude. Using responses to a prompt that showed small gender DIF favoring female test takers, we demonstrate a corpus-based approach that helps address DIF interpretation. To provide linguistic insights into the possible sources of the small DIF effect, this study compared a gender-balanced corpus of 826 writing samples matched by test takers’ performance on the reading and listening components of the test. Four groups of linguistic features that correspond to the rating dimensions, and thus partially represent the writing construct were analyzed. They include (1) sentiment and social cognition, (2) cohesion, (3) syntactic features, and (4) lexical features. After initial screening, 123 linguistic features, all of which were correlated with the writing scores, were retained for gender comparison. Among these selected features, female test takers’ writing samples scored higher on six of them with small effect sizes in the categories of cohesion and syntactic features. Three of the six features were positively correlated with higher writing scores, while the other three were negative. These results are largely consistent with previous findings of gender differences in language use. Additionally, the small differences in the language features of the writing samples (in terms of the small number of features that differ between genders and the small effect size of the observed differences) are consistent with the previous DIF results, both suggesting that the effect of gender differences on the writing scores is likely to be very small. In sum, the corpus-based findings provide linguistic insights into the gender-related language differences and their potential consequences in a testing context. These findings are meaningful for furthering our understanding of the small gender DIF effect identified through statistical analysis, which lends support to the validity of writing scores.

## Introduction

The differences in language use between genders have been studied in various fields and are expected to have social consequences (Mulac et al., 2006). In language assessment, for example, if a subgroup of test takers systematically receives lower scores because of a feature of the test (rather than a true difference in language proficiency), they could consistently be denied access to opportunities, such as admission to an English-medium university. Further, assumptions might develop about what the subgroup can and cannot do that are erroneously attributed to their group membership. Since tests and assessments are widely used as a way to evaluate and compare the achievement or proficiency of test takers and since high-stakes decisions, such as graduation or promotion, are made based on test scores, score users need to be confident that the test items function similarly for all test takers regardless of their backgrounds.

In language testing, disparities in performance by subgroups of test takers are viewed from the perspective of fairness and score validity (Kunnan, 2000; Xi, 2010) and are often explored through differential item functioning (DIF) analysis. Gender-related DIF research has been primarily concerned with whether test takers at the same proficiency level might gain higher scores just because of their gender group membership. Nevertheless, only a few studies have investigated gender DIF in standardized writing tests. Most of them reported the existence of DIF effects favoring female test takers. These effects tended to be small and sometimes negligible. While it has been shown that some DIF findings were consistent across different statistical methods (e.g., Welch and Miller, 1995), none of these effects was triangulated through other sources of data such as the writing samples produced by different gender groups. To the best of our knowledge, this is the first study that has examined the linguistic features of test takers’ writing samples for the prompts flagged as DIF. A motivation of this study is to address the interpretation and explanation of small gender DIF effects of the writing prompts in standardized language proficiency tests, which have been repeatedly reported in the literature. Evaluating the linguistic features of such writing samples provides unique insights into gender-related language differences and their potential consequences in testing contexts. Doing so may also advance our understanding of DIF results in writing assessment.

This study explores the possibility of using corpus analysis tools to examine gender-related linguistic variations in the writing samples elicited by a timed task on a computer-delivered English proficiency test and evaluates the impact of these differences on writing scores. We first survey the literature on gender differences in language use with a focus on writing. We also review gender DIF studies on writing tests, highlighting potential gaps in the research. Next, we describe our study’s research questions, methodology, and results. Finally, we summarize our findings and discuss their implications.

### Gender-Related Language Features

Many studies have discussed and reported gender differences in writing. To understand how the two genders communicate, at the macro-level, Gudykunst and Ting-Toomey (1988) proposed a gender-as-culture hypothesis and described four dimensions of inter-cultural styles. They maintained that generally, women may be perceived as being more indirect in expressing their views, more prone to using sophisticated language, more thoughtful with social roles, and more attentive to others’ feelings in general interpersonal communication. A later empirical study by Mulac et al. (2006) has supported these hypotheses.

At sentence-level, Mulac and Lundell (1994) studied 40 essays written by undergraduate students at a United States university and found that 9 out of 17 language features differed between gender groups. The features associated with male writers included reference to quantity (e.g., over 30,000), elliptical sentences (e.g., to school), and judgmental adjectives (e.g., distracting); while female writers were found to use more of the following features: uncertain verbs (e.g., seems to be), progressive verbs (e.g., processing), locatives (e.g., upper corner of the frame), reference to emotion (e.g., sad), longer mean sentence length, and sentence-initial adverbials (e.g., rather than …, he started …).

In addition to sentence-level features, Jones and Myhill (2007) also examined text-level linguistic features of 718 essays written by secondary school students in the United Kingdom. They reported that the gender differences between the two groups were mainly observed in their frequency of using text-level features, rather than sentence-level features. Their study found that the gender groups differed in their use of 18 out of 35 text-level features. Male students used more topical organization, cohesion as in inter-paragraph linkage, and essay ending features. Female students used more paragraphs and repetition of a proper noun. Meanwhile, only 6 out of 24 sentence-level features were divergent between genders, including sentence length and use of finite verbs. Female students wrote shorter sentences, which is different from the findings in Mulac and Lundell (1994), but they used more finite verbs than male students.

Stylistic differences in writing between the gender groups have attracted attention as well. Rubin and Greene (1992) applied an expanded view of both biological and psychological gender to their study of gender differences in writing at a United States university. They coded multiple stylistic features in samples from 88 students on two types of tasks, namely, expressive/reflective writing and argumentative/extensive writing. Their findings indicated that the stylistic differences were less noticeable between the biological genders compared with the differences across the task types. While the similarity in stylistic features between the gender groups may be conditioned by the task characteristics (e.g., level of formality), Rubin and Greene (1992) found that female writers showed higher excitability with more exclamation points, and a lower level of confrontation with greater consideration for opposite views. The psychological gender roles, which were measured by a psychological role orientation scale, were found to have limited effects.

The exploration of possible linguistic features that are gender-specific has also been approached from a computational perspective. For example, Argamon et al. (2003) analyzed 604 documents from the British National Corpus (BNC) for gender-related differences in fiction and non-fiction genres. They employed machine learning techniques to screen a large number of topic-independent linguistic features (list of function words and list of part-of-speech n-grams), and obtained a set of features that can help identify author gender. Argamon et al. (2003) reported that female works appeared to be more involved since they used first- and second-person pronouns more frequently, while male works contained more informational features with greater numbers of nouns and prepositions and higher type-token ratios.

Overall, these studies suggest that differences in the language used by males and females can be observed in at least four groups of linguistic features, including sentiment (e.g., reference to judgmental adjectives, discussed in Mulac and Lundell, 1994), syntax (e.g., sentence length, discussed in Jones and Myhill, 2007), cohesion (e.g., text-level features, discussed in Jones and Myhill, 2007), and lexical features (e.g., determiners and pronouns, discussed in Argamon et al., 2003). In this study, we focused on these four groups of features to evaluate the effect of gender-specific linguistic differences on test scores. Indeed, syntax, cohesion, and lexical features are commonly used in scoring rubrics to evaluate writing performance (Weigle, 2002). When a writer’s tone and his/her task fulfillment are evaluated in a writing task, sentiment-related features may contribute to the overall evaluation of the writing quality as well. Together, these four groups of features partially represent the construct assessed by the writing test of interest. More information about the correspondence between the feature groups and the scoring rubric is provided in section “Materials and Methods.”

The salience of gender-related language differences may be influenced by contextual factors (Rubin and Greene, 1992). For example, Leaper and Robnett (2011) found that the settings where language examples were elicited tend to influence the observed magnitude of gender language differences, with research lab setting having more pronounced differences. Likewise, gendered differences in writing performance may be influenced by testing conditions such as time constraints and communication modes as studies have shown that test takers’ writing quality in standardized tests may differ from their performance on untimed writing tasks (Riazi, 2016). Nevertheless, studies on gender-related variation in writing have rarely been done in the standardized testing context. When they are, language proficiency, which is another factor that affects the linguistic features produced by test takers, is typically not controlled for. If test takers with the same writing ability have a different probability of receiving the same score on a writing test because of their gender, it will raise concerns about score validity and test fairness. Such concerns are often investigated using DIF methods.

### Gender DIF in Writing Tests

In reviewing the writing DIF literature, we observed three emerging issues: (1) studies investigating gender DIF in writing tasks are rare; (2) where they exist, the gender DIF studies identified large numbers of DIF writing prompts with small effect sizes; and (3) there is a paucity of explanations for the gender DIF patterns observed. We elaborate on these three issues in the following paragraphs.

#### Rarity of Gender DIF Studies in Writing

First, gender DIF has been insufficiently studied on writing tasks compared to other language skills, such as listening and reading, which are often evaluated through multiple-choice items (Zwick et al., 1993). This may be related to the inherent challenges in conducting DIF analyses on writing tests (Welch and Miller, 1995; Chen et al., 2020). One such challenge is the lack of an internal matching variable that could be used in conventional DIF methods to approximate test takers’ proficiency levels (e.g., the corrected total score in a test consists of multiple-choice items).

For DIF studies on writing tests, external matching variables have often been used, either in conjunction with writing scores or without them. For example, to investigate DIF on an eighth-grade assessment of writing skill, Welch and Miller (1995) used three matching variables that are created based on scores of different test components of writing skills, namely, multiple-choice questions only, multiple-choice questions and one writing prompt, and multiple-choice questions and two writing prompts. Gender DIF was identified under all three conditions, and the DIF effects appeared weaker when writing prompt(s) was included to create the matching variables. In their study of TOEFL computer-based test (CBT) writing prompts, Breland and Lee (2007) created an English language ability variable by summing up the standardized scores from three multiple-choice question sections, namely, reading, listening, and structure to examinee gender DIF effect.

Similarly, Chen et al. (2016), whose research this study extends, used multiple external matching variables to investigate gender DIF for the Canadian English Language Proficiency Index Program General (CELPIP-General) writing tasks. They matched test takers on their reading and listening scores rather than on their writing scores. This is because the typical small number of writing tasks on a test, two in their case, limits the usefulness of writing scores as an internal matching variable. For example, if one of the writing prompts is investigated for DIF, then an individual’s writing proficiency will be solely relied on his/her performance on the other prompt. Also, both the reading and listening scores were highly correlated with the writing scores (*r* = 0.80 and 0.73, respectively), which enables using them as covariates to account for the effect of different writing proficiency levels.

#### Prevalence of Small Gender DIF Prompts Favoring Females

Second, in writing DIF studies, it is common for a relatively large number of prompts to be flagged as DIF prompts favoring female test takers but with small effect sizes. Welch and Miller’s (1995) study highlighted that under all three matching conditions, gender DIF effects were consistently present in all six writing prompts and female test takers always had a better chance of receiving higher scores. Similar patterns have been reported by Breland and Lee (2007). They found that among the 87 prompts, 86 were flagged with statistically significant uniform DIF effects and 17 with non-uniform DIF effects. All the DIF prompts favored female test takers, although the effect sizes were “negligible.” Broer et al. (2005) reported a DIF study on the argument and issue prompts in the Graduate Record Examination (GRE). They identified DIF in both types of prompts, with females slightly outperforming their male counterparts. Chen et al. (2016) reported a similar pattern: 29 writing prompts showing gender DIF from a pool of 82, 28 of which favored female test takers with small effect sizes. The directionality and magnitude of gender DIF were similar in all these studies, however, the interpretation of these small DIF effects remains unclear and is worth further investigation.

#### Lack of Methods to Interpret and Explain Gender DIF

Closely tied to our last point, the third issue is a lack of an effective approach to explaining the occurrence of DIF writing prompts. As a statistical inference, the results of a DIF analysis may be influenced by other statistical artifacts (e.g., large sample size leads to statistically significant results without substantive meaning). Therefore, the interpretation of statistical significance will benefit from further evidence to verify the existence of test bias. While the sources of DIF in objective tests are often linked to item features, in performance-based writing tests or other tests that involve human raters (e.g., peer assessment, Aryadoust, 2016), the potential sources of a DIF effect can be multifactorial. A writing DIF effect may be attributed to the features of the writing task, the rubric, the rater(s), the writing samples, and the interactions between these factors. These diverse sources complicate the manifestation of a DIF effect and challenge the identification of its sources.

Commonly used methods for a follow-up analysis of DIF-flagged items include analysis of test content by experts and think-aloud protocols. These methods either assess the features of an item to identify the content-related source of DIF or focus on test takers’ use of cognitive skills in their responding process to determine how it could relate to the DIF effect (Pae, 2011). It should be noted that expert judgments may not be as effective as hoped in explaining the sources of DIF items (Ferne and Rupp, 2007). In an age-based DIF study of the listening items on the Certificate in Advanced English (CAE), Geranpayeh and Kunnan (2007) invited five content experts to judge the potential levels of advantage of an item for each of three age groups. Out of 32 items, the expert panel rated seven items as potentially favoring certain age groups. However, three items (out of seven) matched the DIF items identified statistically and only one item was correctly judged regarding DIF directionality. Geranpayeh and Kunnan (2007) concluded that “expert judges could not clearly identify the sources of DIF for the items” (p. 207).

Test takers’ think-aloud data are another important tool to explore the causes of DIF tasks (Ercikan et al., 2010). However, the effectiveness of this method is highly dependent on test takers’ language proficiency and their ability to verbalize their thinking processes (Alderson, 1990). For example, it may be difficult for highly proficient test takers to realize the automatic processes, such as recognizing a familiar word or phrase.

Besides these methods, an analysis of the linguistic features of writing samples, although rarely used as a follow-up, could be a viable way to investigate the DIF phenomena. Since writing tasks elicit ample linguistic data, the resulting corpus could provide new evidence for validation efforts and studies of fairness (Park, 2014; Xi, 2017). Indeed, it is desirable to take advantage of the advances in corpus linguistics and use corpus-based analysis to evaluate writing DIF. In light of the findings on gender-related language features, the DIF effects identified by analyzing the test scores can be corroborated or refuted with additional evidence from a corpus-based comparative analysis of the essays written by the gender groups matched in the same fashion.

In summary, scholars agree that males and females tend to write differently (Mulac et al., 2006). However, only a handful of DIF studies have investigated gender-related performance differences in writing tests, and no research has examined the extent to which differences in the writing scores can be attributed to gender-related differences in language use. This corpus-based study focuses on an e-mail writing task that demonstrated gender DIF favoring female test takers slightly in Chen et al.’s (2016) writing DIF study and aims to address the following research questions.

Q1. Does the writing of the two gender groups differ in the four groups of construct-related linguistic features, i.e., sentiment and social cognition, cohesion, syntax, and lexical features?
Q2. Do the linguistic differences, if they exist, help explain the divergent scores of males and females on the writing task?

## Materials and Methods

### The Writing Prompt

The writing prompt under investigation is from the CELPIP-General, a general English language proficiency test whose scores are aligned to the Canadian Language Benchmarks (CLB) (Centre for Canadian Language Benchmarks (CCLB), 2012). The test is delivered on a computer. The writing component measures an individual’s ability to effectively use written texts to express ideas, influence others, and achieve other communicative functions in social and workplace contexts. The writing test comprises two tasks. The first is an e-mail to a service provider and the second is a response to a survey question asking for an opinion. In the writing test, test takers are supported with a built-in spell checker.

Each writing sample is evaluated by at least two certified raters independently. The scoring rubric assesses four dimensions of the writing construct: coherence and meaning, lexical range, readability and comprehensibility, and task fulfillment (Paragon Testing Enterprises, 2015)^1^. Task- or prompt-level scores are calculated based on the analytic rubric, averaging across raters. The final writing scores are converted and reported on an 11-level scale (M, 3–12), which corresponds to the CLB levels 1–2 (M) and 3–12.

This study focuses on an e-mail task that was flagged as a uniform DIF prompt favoring female test takers with a small effect size (Chen et al., 2016). The selected writing prompt represents one of the common communication functions elicited in the CELPIP-General test, namely, complaining. This prompt asked test takers to write an e-mail of 150–200 words to a restaurant. They were required to describe their recent visit, to complain about the unavailability of menu options to satisfy their dietary restrictions, and lastly, to suggest solutions.

We selected only one DIF writing prompt to rule out prompt effects on the corpus analysis results because different prompts are likely to elicit writing samples of different linguistic features (Weigle, 2002). Although some dimensions of the writing samples are likely to be comparable across prompts (e.g., lexical range), others (e.g., task fulfillment) are probably different depending on communication goals. For example, the writings of the same proficiency level may have different linguistic features depending on whether the communication goal is to effectively complain about a failed service or to offer advice to a friend. Additionally, different prompts tend to have different magnitude, or even direction, of gender DIF effects. Thus, combining writing samples from different prompts may obscure the interpretations of the corpus analysis. Since the prompt was flagged as favoring females with a negligible effect size (R^2^ change <0.02), we expected that the impact of gender on linguistic features may not be strong. It was nevertheless chosen as an example because this type of gender DIF has been repeatedly reported in the literature (e.g., Welch and Miller, 1995; Breland and Lee, 2007; Chen et al., 2016). The findings of this study may shed light on the occurrence of such gender DIF in standardized writing assessment.

### The Corpus

To remain consistent with the DIF methodology used by Chen et al. (2016), the writing samples were selected based on test takers’ reading and listening scores. Similar to the practice in Breland and Lee (2007) and Chen et al. (2020), the reading and listening scores are used to represent overall language proficiency, which is then used to approximate the writing proficiency to overcome the issue of lacking reliable internal matching variables within a writing test. The corpus comprised 826 writing samples (413 female and 413 male test takers), matched by reading and listening component scores.^2^ The total number of running words in the corpus is 156,474. On average, the female test takers produced longer pieces. Although the difference was statistically significant, the effect size is small (Cohen’s *d*_male–female_ = −0.113, *p* < 0.001). Besides, most of the linguistic features investigated in this study are normalized, making them less likely to be distorted by the text length.

As Table 1 indicates, when reading and listening scores are matched, more male test takers are at the lower writing proficiency bands, which is consistent with the DIF result, i.e., compared to male test takers with similar language proficiency levels, females tended to achieve slightly higher writing scores on this prompt. Recall that this prompt is flagged as showing gender DIF favoring female test takers slightly, the differences in the writing scores between gender groups *cannot* be directly interpreted as “true” proficiency differences, rather it might be seen as a result of matching test takers on their English language proficiency (i.e., reading and listening scores in this case).

**TABLE 1:** Summary of the CELPIP-General corpus of written samples by three writing proficiency bands.

**Gender**	**Level 4**	**Levels 5–8**	**Levels 9–12**	**Number of samples**	**Number of words**
Male	49	281	83	413	76,855
Female	20	306	87	413	79,619

### Selected Linguistic Features and Analytical Tools

Recent development of natural-language-processing (NLP)-based tools has provided new affordance for analyzing writing performance data. In this study, we made use of such tools developed by Kyle and his colleagues as many of their tools have been validated with empirical data by the developers and other researchers.^3^ Informed by the writing construct of the CELPIP-General test (see Paragon Testing Enterprises, 2015) as well as the findings on gender-related writing features, we explored four groups of linguistic features—sentiment and social cognition, cohesion, syntactic features, and lexical features in this study. Table 2 presents how these groups of features could partially represent the scoring dimensions. While these linguistic features are directly or indirectly related to the scoring dimensions, it is worth mentioning that each scoring dimension is more than the sum of the individual linguistic features from the analytical tools. For example, while sentiment and social cognition features may be relevant to the aspects of relevance and tone of the task fulfillment dimension, the same dimension is also concerned with the completeness of responses.

**TABLE 2:** Summary of analytical tools and relevance of the linguistic features to the CELPIP-General scoring dimensions.

**Scoring scale dimensions**	**Feature groups analyzed**	**Number of features analyzed**	**Tool (feature categories)**
Task fulfillment: relevance/tone	General Inquirer (GI)–based indices	34	*SEANCE 1.05* (sentiment/social cognition)
	NRC Word-Association Emotion Lexicon (EmoLex)–based indices	4	
Coherence and meaning: organization	Adjacent lexical/semantic overlaps at sentence level	7	*TAACO* (cohesion features)
Readability and comprehensibility: transitions	Rhetorical connectives	17	
	Repeated words	2	
Readability and comprehensibility: grammar	Clause-based complexity indices	11	*TAASSC 1.4* (syntactic features)
	Noun phrase–based complexity indices	15	
	Usage-based syntactic sophistication indices	12	
	Indices from the L2 Syntactic Complexity Analyzer (L2SCA)	2	
Lexical range: natural use of vocabulary	Frequency of words and n-grams (BNC)	5	*TAALES 1.0* (lexical features)
	Range of words and n-grams (BNC)	7	
	MRC psychological properties of words	3	
	Type token ratios (TTR)	4	*TAACO* (lexical features)

*NRC, National Research Council (Canada); BNC, British National Corpus; MRC, Medical Research Council.*

By selecting features that are valued in the rubric, we could tap into the targeted writing construct because the rating rubric is an operationalization of the abstract construct (Weigle, 2002). The four groups of linguistic features are measured by Sentiment Analysis and Cognition Engine (SEANCE) 1.05 (Crossley et al., 2017), Tool for the Automatic Analysis of Cohesion (TAACO; Crossley et al., 2016), Tool for the Automatic Analysis of Syntactic Sophistication and Complexity (TAASSC) 1.0 (Kyle, 2016), and Tool for the Automatic Analysis of Lexical Sophistication (TAALES) 1.4 (Kyle and Crossley, 2015).

Sentiment and social cognition features were assessed by SÉANCE. We selected a range of features including the individual indices from two sentiment dictionaries, the Harvard IV-4 dictionary–based General Inquirer (GI) and National Research Council (NRC) Word-Association Emotion Lexicon (EmoLex), plus 20 composite indices (Crossley et al., 2016). The GI dictionary is chosen for its comprehensiveness in representing both sentiment and social cognition. It is one of the earliest sentiment dictionaries and is still widely used in research. The GI contains 119 word lists representing 16 categories of emotion and social cognitions.^4^ Social cognition refers to the cognitive processes related to other people and social situation. EmoLex is a newer list of words annotated for eight emotions (anger, fear, anticipation, trust, surprise, sadness, joy, and disgust) and two sentiments (negative and positive). Note that, when judging for sentiment polarity, SEANCE takes the negation markers into account.

The cohesion features were provided by TAACO. We selected the features related to adjacent overlaps of lexical items at sentence level, rhetorical connectives (e.g., basic connectives and the connectives showing rhetorical functions), and occurrence of repeated words. Meanwhile, we excluded paragraph-level adjacent overlap, mainly because the writing samples in the corpus are short, with an average length of 189 words, and many of them are written as a single paragraph.

The syntactic features were captured by TAASSC. We selected various features belonging to the subgroups of the L2 Syntactic Complexity Analyzer (L2SCA) outputs; clause-based complexity indices; noun phrase–based indices; and sophistication indices that focus on verb-argument constructions (VACs, i.e., the units consisting of a verb plus its argument).

To obtain the lexical features, we used both TAALES and TAACO. From TAALES, we selected the features that are calculated with reference to the written corpora such as the written registers in BNC and the Corpus of Contemporary American English (COCA). Also, we utilized word-information-score features based on the Medical Research Council (MRC) Psycholinguistic Database (familiarity, concreteness, imageability, and meaningfulness). Additionally, we paid attention to the type token ratio-based indices from TAACO.

### Data Analysis

The selected variables were further screened in the following manner. We removed the indices that demonstrated extremely low variation (SD < 0.005) or contained a large proportion of zeros (≥80%) because they are not widely represented in the corpus data. Then, to identify the linguistic features that contribute to writing scores, we conducted correlation analyses to identify those showing statistically significant correlations with writing performance (*p* < 0.05). Next, we checked redundancy among the indices to reduce the number of similar features that are *not* statistically different from each other. When two or more indices were closely related (*r* > 0.90), we kept the one with the highest correlation with writing performance. After applying these selection criteria, a total of 123 features were retained, including 38 sentiment and social cognition features, 26 cohesion features, 40 syntactic features, and 19 lexical features.

Considering the non-normal distributions of the majority of the linguistic features, we adopted the Mann–Whitney U tests, the non-parametric counterpart of the independent-sample *t*-test, to assess the differences between male and female test takers. Given the relatively large number of linguistic features investigated in this study, we applied Bonferroni adjustment to the significance levels to better control the overall Type I error rate. The alpha values were adjusted to 0.001 for the sentiment and social cognition features (i.e., 0.05/38) and syntactic features (i.e., 0.05/40), 0.002 for cohesion features (i.e., 0.05/26), and 0.003 for lexical features (i.e., 0.05/19).

We chose to compare the individual features between the gender groups, rather than generating latent variables or components via factor analysis (FA) or principal component analysis (PCA) for the following reasons. First, we are primarily interested in pinpointing measurable differences in specific features that would allow us to compare the findings with previous studies in different contexts. An approach evaluating each language features one at a time is suitable to address our first research question. Likewise, we did not choose FA or PCA to aggregate the variables because the resultant factors or components could be difficult to interpret (e.g., the interpretation may be subjective and makes the results less transparent) and may not well represent all the individual linguistic features.

## Results

This section presents the linguistic features that are statistically significantly correlated with writing proficiency levels and are distinctive between the two gender groups in the four categories, i.e., sentiment and social cognition, cohesion, syntactic features, and lexical features.

### Sentiment and Social Cognition Features

None of the 38 linguistic features in this category showed statistically significant differences between the gender groups at our pre-set significance level (α = 0.001). While female test takers consistently outscored male test takers in most of the features, the effect sizes of gender differences in these features were extremely small (absolute values of Cohen’s *d* ≤ 0.08).

### Cohesion Features

As Table 3 demonstrates, 2 out of 26 cohesion features displayed statistically significant differences between the gender groups. Of the two, one concerned the use of coordinating conjunctions (e.g., “and,” “but,” and “or”), and the other was related to the use of pronouns. Nevertheless, the differences were small in magnitude (Cohen’s *d* ranges from −0.11 to −0.14), with the female test takers having higher scores in both features. That is, the female test takers tended to use more coordinating conjunctions and had a higher ratio of pronouns to nouns in their writing samples. Note that these two features were negatively correlated with writing proficiency, indicating higher values on these cohesion indices are associated with lower writing scores.

**TABLE 3:** Distinctive cohesion features between the two gender groups.

**Features**	**Mann–Whitney U test (male vs. female)**		**Correlation with writing proficiency level**	
	
	**Effect size**	***p***	***r***	***p***
Ratio of pronouns to nouns	−0.140	<0.001	−0.307	<0.001
Number of coordinating conjunctions	−0.110	0.002	−0.129	<0.001

### Syntactic Features

Four out of forty syntactic features were statistically different between the two gender groups. They fall into three categories: noun phrase–based indices [possessives per direct object (no pronoun)], clause-based indices (complex nominals per clause and undefined dependents per clause), and usage-based syntactic sophistication indices (delta P scores). On average, the female test takers outscored their male counterparts in all seven features with small effect sizes (Cohen’s *d*: −0.115 to −0.152).

Table 4 suggests that the female test takers used more sophisticated structures than their male counterparts. The noun phrase–based complexity index that does not count pronouns as part of noun phrases, i.e., possessives per direct object (e.g., “to accommodate my dietary requirements”) was higher for the female test takers. This phenomenon is related to the earlier observation that females used more pronouns in general. The same pattern was observed in the two clause-based syntactic indices, namely, complex nominal per clause (e.g., “Even being able to find options within the menu …”) and undefined dependents per clause (i.e., ungrammatical clauses).

**TABLE 4:** Distinctive syntactic features between the two gender groups (TAASSC).

**Features**	**Mann–Whitney U test (male vs. female)**		**Correlation with writing proficiency level**	
	
	**Effect size**	***p***	***r***	***p***
Possessives per direct object (no pronoun)	−0.129	<0.001	0.143	<0.001
Complex nominals per clause	−0.129	<0.001	0.118	0.001
Delta P scores (verb-construction, SD)	−0.115	0.001	0.112	0.001
Undefined dependents per clause	−0.152	<0.001	−0.324	<0.001

As for syntactic sophistication features that are related to the association strengths of verb argument constructions in reference to COCA written registers, the two gender groups showed some difference in the standard deviations (SD) of delta P scores. Delta P score is a metric of directional strength of association between a verb and a construction with one serving as a cue and another as an outcome or vice versa. A higher value of the SD of the association strengths indicates that females had a larger variation in delta P scores in their use of VACs.

### Lexical Features

None of the 19 lexical features was found to diverge between the two gender groups based on the statistical criterion we set (i.e., α = 0.003). The absolute values of the effect sizes, as measured by Cohen’s *d*, for these indices were smaller than 0.07.

### Summary

The results showed that gender-related writing differences existed in two out of four categories of linguistic features that we explored in this study, namely, cohesion and syntactic features. However, these differences were relatively small, both in terms of the number of statistically significant features and the magnitude of the differences as shown by the effect sizes. Table 5 shows that out of 123 language features compared across gender groups, only six (about 5%) were significantly different. Of the six significant features, three were positively correlated with higher writing scores and the other three were negative, indicating the impacts of these distinctive features on writing scores are in mixed directions and, when presented in one writing sample, their effects on the writing scores could potentially be canceled out. For example, when a writing sample by a female test taker has higher values on all these six features than the one composed by a male test taker, their writing scores are not necessarily different from each other because of the mixed directions between the language features and writing scores. The overall effect of the gender differences on the writing scores may be attenuated with a balanced distribution of correlation directionalities.

**TABLE 5:** Summary of linguistic features studied for the gender groups.

**Feature category (*tool*)**	**Number of features significantly correlated with writing scores**	**Number of features significantly different between gender groups**	**Number of gender-distinctive features that is positively correlated with writing scores**
Sentiment and social cognition (*SEANCE*)	38	0	0
Cohesion (*TAACO*)	26	2	0
Syntactic (*TAASSC*)	40	4	3
Lexical (*TAALES* and *TAACO*)	19	0	0
Total	123	6	3

## Discussion

In this study, we applied a corpus-based analysis to examine an identified gender DIF effect and to investigate its potential linguistic sources. Using test takers’ writing samples, we explored the gender DIF prompt through comparisons of multiple language features across male and female test takers, who were matched on listening and reading scores. The results show that, in standardized writing assessment, gender differences in language use are only observed on a small number of linguistic features and the magnitude of such differences is low. When presenting together, the effects of the gender-specific linguistic features on the writing scores are likely to be attenuated because the direction of these effects is mixed (i.e., some features positively affect the score outcome, while others negatively affect the score outcome). Consistent with the previous statistical analysis results (Chen et al., 2016), the findings of this study suggest that this particular writing prompt was not a serious fairness concern. This confirmation serves as an additional piece of evidence relates to test fairness and contributes to a validity argument for the test scores (Kunnan, 2000).

In interpreting the findings, some cautions are worth noting. First, the results of this study reflect the minimal gender DIF effect observed. Indeed, more substantial differences in linguistic features might be observed between the gender groups if the same analyses were to be conducted on a prompt with a large DIF effect. Similar to the results reported in other previous studies, none of the prompts reported by Chen et al. (2016) was associated with a large effect size. This is, of course, to be expected; tasks in a high-stakes context undergo rigorous review and field testing for fairness before they are used operationally. While using DIF prompt with a small effect size may seem as less optimal for studies that aim to explore gender differences, still, this type of study is helpful in addressing the interpretation of statistically flagged DIF items, especially considering the prevalence of writing prompts that were reported slightly favoring female test takers in different exams (e.g., Welch and Miller, 1995; Broer et al., 2005; Breland and Lee, 2007).

Additionally, while using a single prompt helped us focus on the gender-related features in complaint e-mail writing, the generalizability of the findings to other writing tasks may be restricted, as certain distinctive linguistic features may be prompt specific. For example, emotion-laden lexis and words about social cognition may be less important in a neutral inquiry e-mail than in a complaint. Future studies can investigate whether our results apply to other types of writing prompts.

Furthermore, we acknowledge that, with a large number of hypothesis testing, the possibility of observing difference by chance (i.e., the overall Type I error rate) increases. To find a balance between construct representation and number of linguistic features to be investigated, we have focused on those theoretically related to the targeted writing construct and further reduced the number of features for comparison by excluding those not varying across the writing samples or not contributing to the writing scores. Also, we reported the effect sizes to assist the interpretability of the results. We hope this study provides a first step to looking into gender DIF effect through the lens of the linguistic features of writing samples. Based on the findings of the present study, future studies could test more specific research hypotheses or focus on some of the identified indices to better control the overall Type I error rate.

Finally, it is important to be aware that the DIF effect of a writing prompt can be attributed to a number of factors, such as the prompt, the rubric, the raters, the test takers, the test setting, and the interactions of these factors. Previous studies have focused on the features of prompts (Breland et al., 2004) and the effects of raters (Lumley, 2002); the present study has provided a new angle—the linguistic features of writing samples—to seek for explanations of the DIF effect flagged by statistical methods. Future research could look into how other factors and their interactions may lead to a DIF effect in writing tests. Such investigations will extend our understanding of potential sources of DIF, which go beyond the item and test features.

Despite these interpretive considerations, our findings showed that the responses by female and male writers to the same prompt can differ in a limited number of linguistic features. The manifestation of the gender differences, however, is found to be varying across linguistic features. This implies that test developers and users should be aware of the “value statement” brought in by a rating rubric. Depending on which linguistic features are valued in a rating rubric, the scores may be potentially biased against a gender group. For example, if cohesion features are disproportionately privileged in a rating rubric, compared with another scale that is balanced between cohesion and syntactic features, then, this rubric is more likely to induce gender-related DIF effect. Overall, the combination of corpus-based analysis and quantitative DIF methods can be a valuable addition to more traditional approaches to detecting sources of DIF effects. In the following paragraphs, we discuss the findings in relation to the two research questions.

### Gender-Related Differences in Writing Features

The first research question concerns gender-related differences in language use on a DIF writing prompt. The results regarding the four categories of language features confirmed some of the previous findings and added new insights into gender differences in writing.

Among the language features explored in this study, some cohesion and syntactic measures showed gender differences. In terms of cohesion, the variations between the gender groups appear in one feature of connectives and one pronoun-related feature. These characteristics, to some extent, echoed the differences found in the previous studies (Rubin and Greene, 1992; Argamon et al., 2003). The e-mails written by the female test takers in this study outscored those written by the male test takers on both cohesion indices, suggesting that the writing of the females was more cohesive through more frequent uses of more coordinating conjunctions and pronouns. Nevertheless, as pointed out by a reviewer, using more coordinating conjunctions and pronouns does not necessarily make the writing samples more coherent. Overly relying on such explicit cohesive devices may add redundancy and make the writing unnatural. Indeed, the negative correlations between the two cohesion indices and writing scores suggest that highly proficient writers are less likely to rely on these features to achieve coherence.

Four syntactic features were found to be different between gender groups with the female test takers outscored their male counterparts on all four features. This trend toward more sophisticated language is somewhat consistent with the general perception of female writing (Gudykunst and Ting-Toomey, 1988). Our findings demonstrate that the female test takers packaged more information at the noun phrase and clause levels with more frequent use of structures like multiple complex nominals per clause. However, Rubin and Greene (1992) noted that writers with a masculine gender role orientation tended to use more complex sentence structures, which contradicts the evidence of this study. The contradiction may be explained by the difference in the writing genres (university academic writing tasks vs. personal e-mails). We also found that the female test takers’ e-mails had a larger SD in delta P scores, which suggests that female test takers used structures that showed a larger variability in the strength of association, as measured in delta P scores in reference to all the written corpora in COCA. This may help clarify that the sophistication levels of the language used by all test takers were reflected in their adoption of the more common VAC structures or lexical items employed by native speakers of English (Kyle, 2016).

The present study did not identify gender-related differences in lexical, sentiment, and social cognition features. Although statistically non-significant, the writing of the female test takers showed marginally lower lexical sophistication, with higher MRC familiarity scores as well as higher scores of word frequency and more regular use of trigrams in reference to the BNC written registers.

With regard to sentiment and social cognition features, previous studies suggested that female test takers tended to use more reference to emotion and judgmental adjectives (Mulac and Lundell, 1994) and employ more personal pronouns to refer to themselves, which tends to render their writing more narrative (Rubin and Greene, 1992). Although these differences were not statistically significant in this study, we observed similar patterns showing that female test takers were slightly more likely to use personal pronouns that refer to themselves and use emotion-related words for both negative and positive feelings.

Considering the large number of linguistic features analyzed, the proportion of those that were distinctive is rather small. Some of these features have been confirmed in previous studies (e.g., use of pronouns), while new ones may be considered in future studies on gender-related linguistic features. However, we need to bear in mind that some of the linguistic features identified as distinctive may be more relevant to the writing task (e-mail writing) or environment (writing on a computer and under a time constraint) in this study.

### Language Differences and Writing Performance

The second question concerns whether the identified gender-related linguistic features contributed to divergent writing performance between the gender groups. The correlational information between these features and the CELPIP-General writing levels sheds light on the small gender DIF effect observed on the writing scores.

Most of the relationships between the gender-related language features and writing performance are in line with theoretical expectations of the writing construct (see Table 2). We hypothesized that the sentiment and social cognition features would contribute to performance on the CELPIP-General writing test with regard to task fulfillment, which includes the relevance of the content, completeness, tone, and length of the text (Paragon Testing Enterprises, 2015). The significant correlations were found in both directions; that is, some sentiment and social cognition features were positively correlated with higher writing scores (e.g., negative sentiment), while others were negative (e.g., positive sentiment and first-person pronouns). Recall that the task was writing a complaint e-mail, the correlations between these features and the writing scores were consistent with our expectation. However, none of them was statistically different across gender. Similarly, although some lexical features were associated with writing scores (e.g., word frequency, trigram), none was significantly divergent between gender groups.

In the two groups of features, cohesion and syntactic features, where gender differences were observed, the two cohesion features, which pertained to the number of coordinating conjunctions and pronouns, were negatively correlated with the writing scores. This pattern of the correlation is consistent with findings of Crossley et al. (2016), where the authors reported that local cohesion (e.g., sentence-level overlaps of verb synonyms) and overall text cohesion (e.g., the pronoun-to-noun ratio and lemma TTR) were negatively correlated with the scores of the essays written on the Scholastic Aptitude Test (SAT) prompts. However, the global cohesion features such as overlaps of certain lexical units (e.g., adverb lemmas, all lemmas, and verb lemmas) among three adjacent paragraphs have been positively associated with writing scores (e.g., Crossley et al., 2016). Also, it has been asserted that features of global cohesion were more predictive of essay quality than local cohesion measures such as the use of connectives (Guo et al., 2013). However, due to the settings of the language proficiency exam, we could not meaningfully report or compare the features measuring cohesion between paragraphs. The large-scale language proficiency test allows test takers limited time to develop their writing responses (27 min in this case), which leads to short writing samples (mean = 189 words and SD = 38.20). In particular, many test takers submitted a single-paragraph writing sample, which is not uncommon for an e-mail writing task.

Among the four syntactic features that are different between the gender groups, three were positively correlated with writing proficiency and one was negatively correlated with it. Although the configuration of correlations was roughly as expected, compared with other studies, a somewhat unique set of features was associated with this particular writing task. Except for one of the positively correlated features that has been reported in previous studies (i.e., complex nominals per clause, see Lu, 2010), all the others were either not investigated directly or were not found to be related to writing scores. Particularly, the possessive pronoun–related feature is unique to this study; this may have reflected the wide use of possessive pronouns in the e-mail samples. Interestingly, for the delta p scores, a feature based on association strength, its variation exerted more influence on the writing scores than the trait itself.

Overall, female test takers consistently outscored their male counterparts on all the distinctive features identified in the present study. These distinctive language features, however, varied in the magnitude and direction of their correlation coefficients (i.e., from −0.324 to 0.143) with writing performance, suggesting that some of these language features contribute to higher writing scores while others are associated with lower scores. These findings imply that the writing construct of this test, as operationalized through the writing task and the rating rubric is not heavily impacted by the clusters of the linguistic features associated with a gender group. When taken together, they may give an edge to the female test takers, whose texts showed more of these features than those of their male peers. However, given the small to moderate effect sizes of the correlation coefficients, the impact of gender-related differences on the scores was probably minimal. Still, it is worth noting that for female test takers whose profile of linguistic features had more occurrences of the positively perceived features and fewer of the negatively perceived, their advantage in writing scores may be more pronounced.

## Conclusion

In sum, this study examined the linguistic features of responses to a writing prompt that was flagged showing small gender DIF favoring female test takers—which is a typical finding in the writing DIF studies. Despite the limitations acknowledged at the beginning of section “Discussion,” this study demonstrated an additional way for further exploring and understanding the DIF results based on statistical analyses of scores. The finer distinction of dissimilar linguistic features in this corpus-based study provides a good opportunity to examine gender-related differences in greater depth. This approach can be used in other writing tests and hopefully, it will help language testers interpret and explain the DIF effects in large-scale standardized writing tests.

## Data Availability Statement

The datasets generated for this study will not be made publicly available as the writing samples analyzed in this study were responses to a high-stakes language proficiency test. The samples and the test materials are the properties of the test publisher, Paragon Testing Enterprises.

## Ethics Statement

Ethics review and approval was not required for the study on human participants in accordance with the local legislation and institutional requirements. The participants provided their written informed consent to share their anonymised responses for research purposes.

## Author Contributions

ZL and MC designed the study, conducted analysis, and wrote the manuscript. JB contributed to the critical revision of the manuscript and assisted with study concept or design.

## Conflict of Interest

The authors declare that the research was conducted in the absence of any commercial or financial relationships that could be construed as a potential conflict of interest. The handling Editor declared a past collaboration with the authors.

## Footnotes

1 See a brief description of the rating rubric under *Writing Performance Standards* at www.celpip.ca/test-scoring/ (accessed in January, 2019).

2 The top three countries of nationality of these test takers were the Philippines (*N* = 192), India (*N* = 135), and China (*N* = 55).

3 Those tools were obtained from http://www.kristopherkyle.com/tools.html.

4 See more details at http://www.wjh.harvard.edu/~inquirer/homecat.htm.
